# Exploring the Potential Use of a PBMC-Based Functional Assay to Identify Predictive Biomarkers for Anti-PD-1 Immunotherapy

**DOI:** 10.3390/ijms21239023

**Published:** 2020-11-27

**Authors:** Silvia M. Bacot, Taylor A. Harper, Rebecca L. Matthews, Christie Jane Fennell, Adovi Akue, Mark A. KuKuruga, Shiowjen Lee, Tao Wang, Gerald M. Feldman

**Affiliations:** 1 Office of Biotechnology Products, Center for Drug Evaluation and Research, U.S. Food and Drug Administration, Silver Spring, MD 20993, USA; Silvia.Bacot@fda.hhs.gov (S.M.B.); tah9h@virginia.edu (T.A.H.); rebecca.matthews@nih.gov (R.L.M.); ChristieJane.Fennell@fda.hhs.gov (C.J.F.); 2 Office of Vaccines Research & Review, Center for Biologics Evaluation and Research, U.S. Food and Drug Administration, Silver Spring, MD 20993, USA; Adovi.Akue@fda.hhs.gov (A.A.); Mark.Kukuruga@fda.hhs.gov (M.A.K.); 3 Office of Biostatistics and Epidemiology, Center for Biologics Evaluation and Research, U.S. Food and Drug Administration, Silver Spring, MD 20993, USA; Shiowjen.Lee@fda.hhs.gov

**Keywords:** Nivolumab, biomarker, peripheral blood mononuclear cells, T cells, cytokines

## Abstract

The absence of reliable, robust, and non-invasive biomarkers for anti- Programmed cell death protein 1 (PD-1) immunotherapy is an urgent unmet medical need for the treatment of cancer patients. No predictive biomarkers have been established based on the direct assessment of T cell functions, the primary mechanism of action of anti-PD-1 therapy. In this study, we established a model system to test T cell functions modulated by Nivolumab using anti-CD3 monoclonal antibody (mAb)-stimulated peripheral blood mononuclear cells (PBMCs), and characterized T cell functions primarily based on the knowledge gained from retrospective observations of patients treated with anti-PD-1 immunotherapy. During a comprehensive cytokine profile assessment to identify potential biomarkers, we found that Nivolumab increases expression of T helper type 1 (Th1) associated cytokines such as interferon-γ (IFN-γ) and interleukin-2 (IL-2) in a subset of donors. Furthermore, Nivolumab increases production of Th2, Th9, and Th17 associated cytokines, as well as many proinflammatory cytokines such as IL-6 in a subset of donors. Conversely, Nivolumab treatment has no impact on T cell proliferation, expression of CD25, CD69, or Granzyme B, and only modestly increases in the expansion of regulatory T cells. Our results suggest that assessment of cytokine production using a simple PBMC-based T cell functional assay could be used as a potential predictive marker for anti-PD-1 immunotherapy.

## 1. Introduction

Although immune checkpoint inhibitors (ICIs) such as anti-PD-1/PD-L1 therapeutic antibodies like Nivolumab and Atezolizumab achieve remarkable benefits for many types of cancer, including melanoma and lung cancer, etc., identification of predictive biomarkers for anti-PD-1 therapy is one of the major challenges in developing effective ICI therapies for cancer treatment. Currently, there are two U.S Food and Drug Administration-approved biomarkers that predict the efficacy of ICI treatment: (1) Expression of PD-L1 in tumor tissues; and (2) the presence of microsatellite-instability–high (MSI-H)/DNA mismatch repair–deficient (dMMR) in tumor tissues. However, many patients with positive expression of these biomarkers do not respond to therapy, while some patients with negative expression of these markers do respond to therapy [1,2,3]. Furthermore, ethical and technical challenges abound regarding the use of these two biomarkers in the clinic, such as requiring sufficient tumor material via invasive and inconvenient approaches, sampling biases that do not reflect tumor heterogeneity, lack of accuracy and reproducibility of laboratory protocols, and analysis of complex genomic alteration data of tumor samples, all of which significantly limit the implementation of these biomarkers in clinical practice [1,2,4]. Lastly, neither of these biomarkers directly assesses treatment-induced T cell responses, which are thought to be the main mechanism of action of anti-PD1 therapy. Consistent with this line of inquiry, analyses of tumor mutation burdens and alterations in DNA damage response and repair (DDR) genes, as well as infiltration of immune cells (among others) have been explored [2,5,6], though they have been met with the same challenges as described for the assessment of expression of PD-L1 and MSI-H/dMMR.

Due to the many challenges in assessing biomarkers in patient tumor tissues, extensive efforts have been made to identify biomarkers using liquid biopsies such as blood cells or serum samples. For example, one promising approach involves the analysis of genetic variations in circulating cell-free DNA (cfDNA) by next generation sequencing. It has been shown that hypermutated circulating tumor DNA may correlate with the patient’s response to immune therapy [7,8]. However, there are many challenges associated with using data from circulating tumor DNA, such as the technical challenge of deep sequencing very low copies of tumor cfDNA. Moreover, the mutation rates in circulating tumor DNA are not always directly reflective of the immune response induced by anti-PD-1 therapy [7,8,9]. Therefore, further validation is required to determine whether the assessment of mutation rates of circulating tumor DNA can be used as a biomarker for anti-PD-1 therapy.

Direct assessment of T cell functions using blood or serum samples has been previously explored to predict the efficacy of anti-PD-1 therapy in the clinic, though to a very limited extent. These limited studies have shown that patient responses to anti-PD-1 therapy are most often positively associated with an increase in frequency or absolute cell count of different populations of immune cells, such as neutrophils and lymphocytes, eosinophils [10], monocytes [11,12,13], CD4^+^ and CD8 T cells [11,12], NK cells [12], and regulatory T cell subsets [13], as well as Ki67 and Granzyme B positive T cells [14,15,16]. In addition, the levels of cytokines, soluble PD-L1, or growth factors in serum have also been shown to be associated with a positive outcome in patients treated with anti-PD-1 therapy [17,18,19,20,21]. However, the patient sample sizes in these studies were very small and prone to contradictory results. For example, increased levels of IFN-γ were observed in some patients who experienced improved responses to Nivolumab (an FDA-approved mAb to PD-1) and Pembrolizumab (also an FDA-approved anti-PD-1 mAb) [22,23,24], while other studies demonstrated no association of serum IFN-γ with the patient response to Nivolumab [17]. Notably, most of these studies were retrospective rather than prospective, such that analysis of the differences in potential biomarkers between pre- and post- treatment samples was not performed. More importantly, these biomarkers do not appear to be directly related to the functions of T cells induced by anti-PD-1 therapy.

Assessing T cell functions modulated by immune checkpoint inhibitors has been used to a limited extent to characterize the effects of the anti-PD-1 mAb Nivolumab on human T cell responses. Such characterization assessments include mixed lymphocyte reaction (MLR) assays, bacteria superantigen SEB (staphylococcal enterotoxin B) stimulation assays, and cytomegalovirus (CMV)-re-stimulation assays [25,26]. Although these assays would be sufficient to characterize T cell responses induced by Nivolumab, they are not appropriate for clinical applications in anti-PD-1 therapy. For example, the MLR assay cannot differentiate T cell functions between individual donors due to the mixing of immune cells from two donors as required for this assay. Similarly, the SEB based assay can only assess the functions of T cells expressing T cell receptor (TCR) V chains, which accounts only for a small percentage of human T cells [25]. Finally, the CMV re-stimulation assay can only assess memory T cell responses to CMV. Overall, the scope of these functional assessments as described above are very limited.

To overcome the limitations of these ex vivo assessments of T cell functions, we conducted a comprehensive analysis of immune responses to Nivolumab using a PBMC based assay, which more closely resembles the in vivo treatment of anti-PD-1 therapy. With the intent to identify potential parameters that specifically respond to Nivolumab treatment, we focused on cytokine profiling due to the existence of reliable and robust cytokine assays which have potential in predicting the efficacy of anti-PD-1 immunotherapy. In addition, we performed flow cytometry analyses to assess expression of the T cell proliferation marker Ki67, the expression of the activation markers CD69 and CD25, and the percentage of inducible CD4^+^CD25^+^FoxP3^+^ regulatory T cells. 

## 2. Results

### 2.1. Nivolumab Significantly Increases Th1 Associated Cytokine Production in a Donor-Dependent Manner

Since expression of PD-1 is required for the effects of Nivolumab on T cell activation, and PD-1 expression is low on both CD4^+^ and CD8 T cells [26], we utilized a soluble cross-linked anti-CD3 mAb to stimulate expression of PD-1 on T cells. We found that anti-CD3 mAb stimulation induces expression of PD-1 in both CD4^+^ and CD8^+^ T cells in all tested donors (Supplementary Figure S1A,B). 

To simplify the analysis of cytokine productions, we categorized them as Th1, Th2, Th9, and Th17-associated cytokines. These categories include cytokines that are either involved in the differentiation of or secretion by these T cell subsets (albeit with some overlap). We also examined the effects of Nivolumab on the expression of other proinflammatory cytokines, such as IL-6, IL-1α, and IL-1β, which are primarily secreted by activated myeloid cells and play critical roles in inflammation.

**Figure 1:**
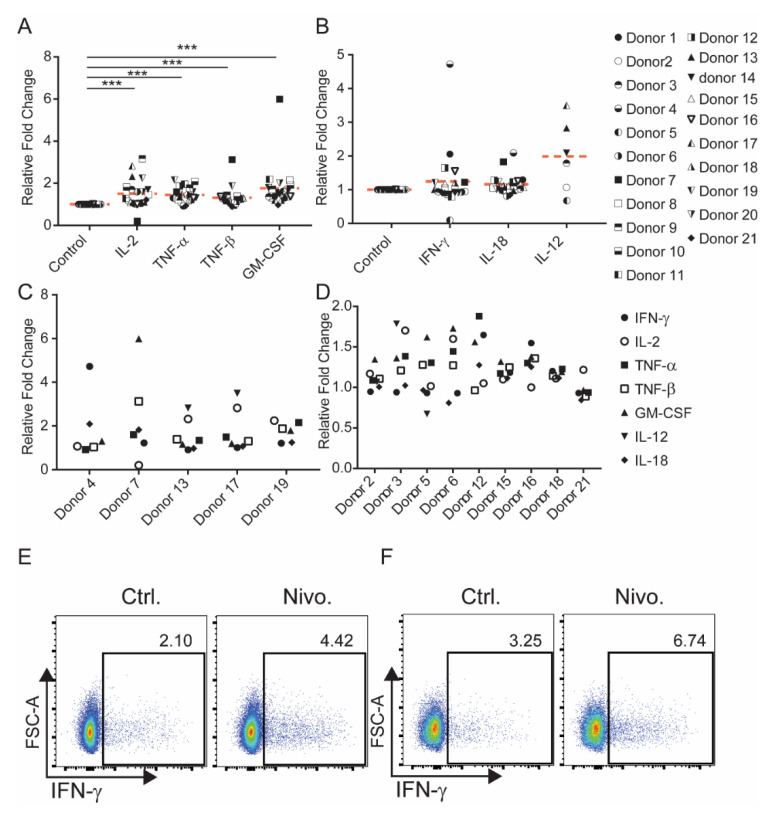
Induction of Th1-associated cytokine production by Nivolumab. (**A**,**B**) Frozen PBMCs from 21 healthy donors were thawed and cultured in RPMI1640 medium containing 5% AB human serum. Cells were then treated with Nivolumab (20 μg/mL) in the presence of anti-CD3 mAb (0.1 μg/mL) for three days. Cell supernatants were harvested and tested for cytokine production by Luminex analyses. (**C**,**D**) Data representing specific donors from Figure 1A are shown. (**C**) Donors whose PBMCs produced at least two cytokines at concentrations greater than two-fold than that of controls in response to Nivolumab treatment are shown. (**D**) Donors whose PBMCs did not produce any cytokines at least two-fold greater than those of the controls are shown. (**E**,**F**) Cell culture was performed as described in Figure 1A,B. Cells were harvested and intracellularly stained with fluorescence-conjugated IFN-γ for flow cytometric analysis. The expression of IFN-γ in CD4^+^ (**E**) and CD8^+^ (**F**) T cells are shown (*n* = 10). *** *p* < 0.001.

In assessing IL-2, TNF-α, TNF-β, and GM-CSF, the extent of the observed increase was highly heterogeneous and donor dependent. The average fold increase over untreated control cells induced by Nivolumab ranged from 1.31 (TNF-β) to 1.78 (GM-CSF) (Figure 1A). When assessing the 21 donors tested individually, two donors showed a two-fold increase in the production of TNF-α (donors 10 and 19), four donors showed a two-fold increase in the production of IL-2 (donors 9, 13, 17, and 19), and four donors showed a two-fold increase in the production of GM-CSF (donors 7, 8, 11, and 20). One donor (donor 7) experienced more than a two-fold increase in the production of TNF-β compared to the control (Figure 1A). Nivolumab treatment did not have a statistically significant impact on the overall production of IFN-γ or IL-18, although two donors showed a greater than two-fold increase in the production of IFN-γ (donors 1 and 4) and one donor showed a greater than two-fold increase in the production of IL-18 (donor 4) (Figure 1B). Similarly, PBMCs from most donors did not produce detectable levels of IL-12, although six donors demonstrated detectable levels of IL-12, and three of them showed a greater than two-fold increase in IL-12 production (Figure 1B). Further analyses indicate that the cytokine profile was also highly heterogeneous. While five donors (donors 4, 7, 13, 17 and 19) showed that production of at least two cytokines can be induced by Nivolumab (Figure 1C), Nivolumab treatment did not result in an increase in any cytokine in nine donors (Figure 1D). The increased expression of IFN-γ by Nivolumab was observed in both CD4 and CD8 T cells by flow cytometric analysis (Figure 1E,F). Among 10 donors tested, Nivolumab increased IFN- γ production in six donors (data not shown), indicating that the increased IFN-γ expression observed by flow cytometry is significantly higher than that observed by the Luminex assay. The discrepancy between the two assays may be due to that IFN-γ production observed in the Luminex assay is also from other types of immune cell, such as NK cells or, conversely, that the cells in the longer culture use up the IFN-γ produced. Of note, Nivolumab treatment in the absence of anti-CD3 mAb treatment had no impact on the production of IFN-γ, IL-2, TNF-α TNF-β IL-12, GM-CSF, and IL-18 in any of the five tested donors (Supplementary Table S1). 

Of note, the magnitude of elevated cytokine production in our PBMC model is lower than that observed in a dendritic and T cell co-culture mixed lymphocyte reaction assay (MLR) [27]. The main mechanism of action of Nivolumab modulating T cell responses is through blocking the interaction of PD-1 expressed on T cells and PD-L1 expressed on stromal cells and cancer cells within the tumor microenvironment. Thus, we hypothesize that the difference in cytokine production levels between the two systems may be due to the differences in PD-L1 expression. Indeed, we found that Nivolumab treatment significantly increased expression of PD-L1 on non-T cells in the PBMC model, but that the expression level of PD-L1 is significantly lower in dendritic cells and T cells used in our co-culture MLR model (Supplementary Figure S2A,B).

To confirm that these increased levels of cytokines upon Nivolumab treatment are derived from T cells, we performed intracellular flow cytometric analyses of induction of IFN-γ gated on T cells. We found that Nivolumab increased IFN-γ production in both CD4^+^ and CD8^+^ T cells (Figure 1E,F).

The effects of Nivolumab are expressed as fold changes instead of raw values of cytokine production to minimize the variation in starting values of each cytokine. This usage of fold change is also convenient for the assessment of effects of Nivolumab treatment in a clinical setting. The raw data of each cytokine tested are provided in Supplementary Table S2.

Taken together, our data demonstrate that Nivolumab treatment increases the production of Th1 associated cytokines in a small subset of donors. The modulation of Th1 associated cytokine production by Nivolumab is donor dependent, and cytokine production in response to Nivolumab among individual donors is highly heterogeneous.

### 2.2. Nivolumab Significantly Increases Th2, Th9, and Th17 Associated Cytokine Productions in a Donor-Dependent Manner

**Figure 2:**
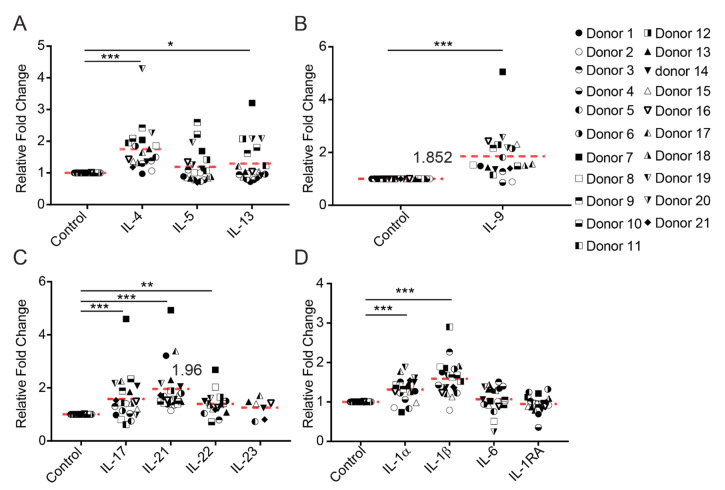
Nivolumab induces production of Th2, Th9, and Th17-associated cytokines in a donor-dependent manner. Frozen PBMCs from 21 healthy donors were thawed and cultured in RPMI1640 medium containing 5% AB human serum. Cells were then treated with Nivolumab (20 μg/mL) in the presence of anti-CD3 mAb (0.1 μg/mL) for three days. Cell culture supernatants were harvested for assessing levels of Th2 (**A**), Th9 (**B**), Th17 (**C**) and other proinflammatory cytokines (**D**). * *p* < 0.05, ** *p* < 0.01, *** *p* < 0.001.

Little is known about whether anti-PD-1 therapy has an impact on the cytokines produced by other types of T cells. We examined whether Nivolumab has an impact on the production of those cytokines associated with Th2, Th9, and Th17 cells [28,29]. We found that, on average, Nivolumab treatment significantly increased the production of the Th2 associated cytokines IL-4 (1.75-fold) and IL-13 (1.29-fold). Nivolumab increased by two-fold the production of IL-4 in five donors (donors 7, 9, 10, 19, and 20), and IL-13 production in four donors (donors 7, 12, 19, and 20), respectively (Figure 2A). While Nivolumab treatment did not significantly increase the overall production of IL-5, two donors showed a two-fold increase over the untreated controls (donors 9 and 10) (Figure 2A).

Treatment with Nivolumab significantly increased the Th9 associated cytokine IL-9, with eight donors showing a two-fold increase compared to the control (donors 6, 7, 9, 12, 15, 16, 19, and 20) (Figure 2B). Nivolumab also significantly increased production of the Th17 associated cytokines IL-17A and IL-21 (Figure 2C), although the effects of Nivolumab on these cytokines were also variable between donors: Nivolumab increased IL-17A production by two-fold in five donors (donors 7, 10, 14, 17, and 19), and increased IL-21 production in six donors (donors 1, 7, 13, 14, 17, and 19), respectively. Of the Th17 associated cytokines, IL-21 production underwent the greatest induction by Nivolumab with an average fold increase of 1.96. While Nivolumab treatment, on average, significantly increased the production of the Th17 associated cytokine IL-22 and two donors showed a two-fold increase compared to control (donors 7 and 8), four donors did not have detectable levels of IL-22 in response to Nivolumab treatment (Figure 2C). PBMCs from only seven donors produced detectable levels of the Th17-associated cytokine IL-23, and no donor showed an increase in IL-23 production (Figure 2C).

We next characterized the cytokine profile modulated by Nivolumab by examining proinflammatory cytokine production of IL-1α, IL-1β, IL-1RA, and IL-6. We found that Nivolumab treatment significantly increased the production of IL-1α and IL-1β, but not IL-6 or IL-1RA (Figure 2D). Two donors showed a two-fold increase in the production of IL-1 β (donors 3 and 11), whereas none showed more than a two-fold increase in the production of IFNα, IL-1α, IL-6, and IL-1RA. Although one donor showed a two-fold increase in the production of IFN-α (donor 6), all other donors had an undetectable level or very low level of production of IFN-α. The cytokines IL-7 and IL-15 were either undetectable or below the limits of detection in any of the donors, whether treated with Nivolumab or not (Supplementary Table S2). Only two donors (donors 5 and 6) had detectable levels of IL-27 (Supplementary Table S2).

A more in-depth analysis of the data showed that some donors tended to respond better to Nivolumab in terms of the types of cytokines produced. For example, eight individual cytokines were induced by more than two-folds by Nivolumab in one donor (donor 7). In contrast, no cytokines were inducible by Nivolumab to any significant extent in three donors (donors 2, 5, and 21) (Supplementary Table S2).

In summary, our data demonstrate that Nivolumab has profound effects on the production of Th1, Th2, Th9, and Th17 associated cytokines, as well as other, proinflammatory cytokines, in a subset of donors, and that the donor-specific responses to Nivolumab treatment are highly heterogeneous.

### 2.3. Nivolumab Treatment Did Not Significantly Affect T Cell Proliferation

**Figure 3:**
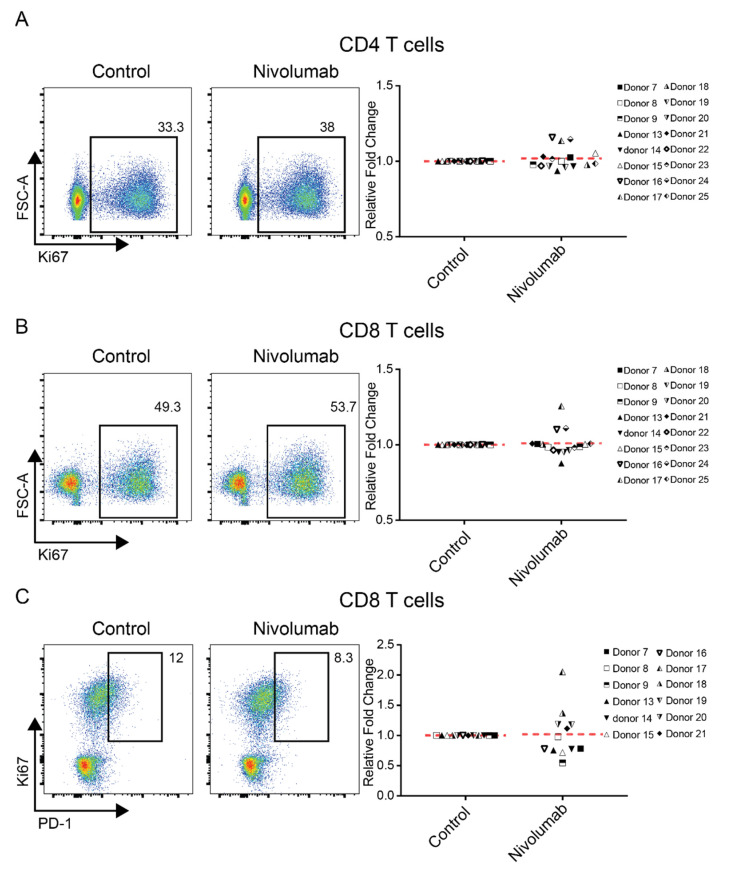
Nivolumab treatment does not impact T cell proliferation. Frozen PBMCs were thawed and cultured in RPMI1640 medium containing 5% AB human serum. Cells were then treated with Nivolumab (20 μg/mL) in the presence of anti-CD3 mAb (0.1 μg/mL) for three days and harvested for flow cytometric analysis of expression of the T cell proliferation marker Ki67 (*n* = 16). Shown is the expression of Ki67 in CD4^+^ T cells (**A**) and CD8^+^ T cells (**B**). (**C**) The expression of PD-1^+^Ki67^+^ on CD8^+^ T cells is shown. The left panels show the data from one representative donor, whereas the right panel provides a summary of data from 12 different individual donors.

Increased expression of the proliferation marker Ki67 in both tumor infiltrating T cells and circulating T cells has been observed in patients treated with anti-PD-1 therapy [15,16]. Increased expression of Ki67 in CD4^+^ and CD8^+^ T cells treated with Nivolumab in vitro was also observed using the MLR assay [27]. We examined whether Nivolumab treatment can enhance T cell proliferation by assessing Ki67 expression in both CD4^+^ and CD8^+^ T cells. On average, Nivolumab treatment did not significantly enhance the expression of Ki67 in either CD4^+^ or CD8^+^ T cells (Figure 3A,B) compared to the control, with no donor showing even a two-fold increase in the expression of Ki67.

Recent studies have demonstrated that the level of increased PD-1 proliferation in CD8^+^ T cells is associated with the efficacy of Nivolumab in lung cancer and other types of cancer patients [30,31]. We examined whether similar results can be observed in our PBMC-based assay. On average, Nivolumab treatment did not significantly enhance the expression of PD-1^+^Ki67^+^ in CD8^+^ T cells (Figure 3C) compared to its control, although Nivolumab did enhance the expression of PD-1^+^KI67^+^ in CD8^+^ T cells by at least two-fold in one donor (donor 17) (Figure 3C).

### 2.4. Nivolumab Does Not Increase the Expression of T Cell Activation Markers CD69 or CD25

**Figure 4:**
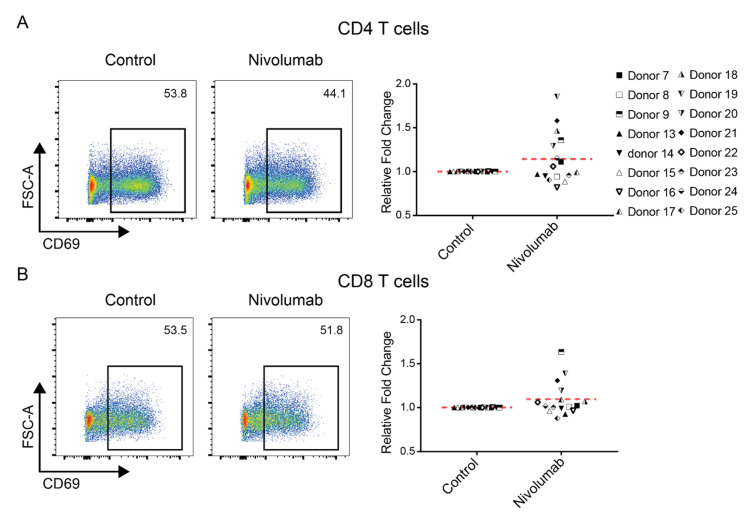
Nivolumab treatment does not impact T cell activation. Frozen PBMCs were thawed and cultured in RPMI1640 medium containing 5% AB human serum. Cells were then treated with Nivolumab (20 μg/mL) in the presence of anti-CD3 mAb (0.1 μg/mL) for three days and were then harvested for flow cytometric analysis of the expression of T cell activation marker CD69. Shown is the expression of CD69 in CD4^+^ T cells (**A**) and CD8^+^ T cells (**B**). The left panels show the data from one representative donor, whereas the right panel provides a summary of data from 16 different individual donors.

Increased expression of T cell activation markers CD69 and CD25 was observed in CD4^+^ and CD8^+^ T cells when treated with Nivolumab during the course of the MLR assay [27]. We tested whether Nivolumab has similar effects using our PBMC-based model. Flow cytometry analysis indicated that three-day Nivolumab treatment did not significantly increase expression of CD69 (Figure 4) or CD25 (Supplementary Figure S3A,B) in either CD4^+^ or CD8^+^ T cells compared to their controls. Similarly, Nivolumab did not significantly affect the expression of the HLA-DR, another T cell activation marker, in either CD4^+^ or CD8^+^ T cells compared to their controls (Figure 2C,D).

Since CD69 is an early T cell activation marker, we also investigated whether a shorter (< 24 h) Nivolumab treatment affects the expression of CD69. Similar to the results observed during three-day Nivolumab treatment, we found that an 18-h treatment with Nivolumab did not have a significant impact on the expression of CD69 in either CD4^+^ or CD8^+^ T cells compared to their controls (Supplementary Figure S4A,B). 

### 2.5. Nivolumab Does Not Impact the Expression of Granzyme B in T Cells

**Figure 5:**
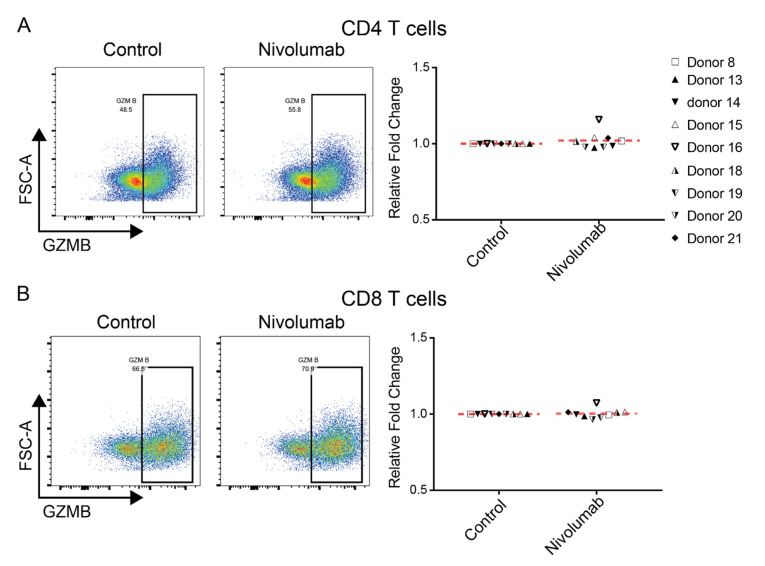
Nivolumab treatment does not impact the expression of Granzyme B. Frozen PBMCs were thawed and cultured in RPMI1640 medium containing 5% AB human serum. Cells were then treated with Nivolumab (20 μg/mL) in the presence of anti-CD3 mAb (0.1 μg/mL) for three days and were then harvested for flow cytometric analysis of expression of Granzyme B. Shown is the expression of Granzyme B in CD4^+^ T cells (**A**) and CD8^+^ T cells (**B**). The left panels depict data from one representative donor, whereas the right panel provides a summary of data from nine different individual donors.

Increased expression of the T cell functional marker Granzyme B is observed in CD4^+^ and CD8^+^ T cells by in vitro Nivolumab treatment [27]. Clinically, the expression levels of Granzyme B in tumor tissues are correlated with patient responses to treatment with both Nivolumab [14] and Pembrolizumab [32]. We tested whether Nivolumab has similar effects in our model system, as demonstrated by flow cytometric analyses. We found that Nivolumab treatment did not significantly affect the expression of Granzyme B in either CD4^+^ (Figure 5A) or CD8^+^ T cells (Figure 5B) compared to their controls.

### 2.6. Nivolumab Induces a Modest Expansion of Inducible Regulatory T Cells

**Figure 6:**
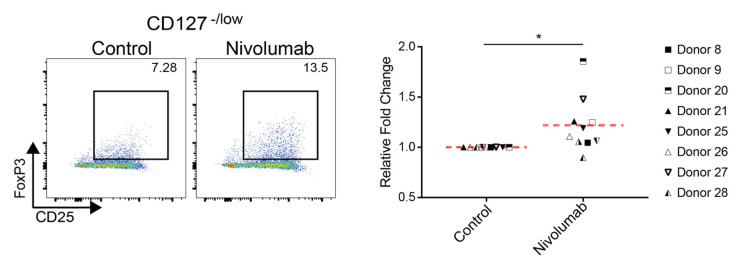
Nivolumab treatment induces the expansion of regulatory T cells. Frozen PBMCs were thawed and cultured in RPMI1640 medium containing 5% AB human serum. Cells were treated with Nivolumab (20 μg/mL) in the presence of anti-CD3 mAb (0.1 μg/mL) for three days and were subsequently harvested for flow cytometric analysis of the expression of regulatory T cell markers CD25 and FoxP3 gated on CD4^+^ CD127^-/low^ T cells. The left panels show the data from one representative donor’s PBMCs, whereas the right panel provides a summary of data from eight different individual donors. * *p* < 0.05.

Regulatory T cells (Tregs) play a critical role in the anti-tumor T cell response, and anti-PD-1 treatment has been shown to induce the expansion of Tregs in tumor tissues. Significantly, a higher percentage of Tregs are positively associated with patient response rates to Nivolumab treatment [13,33]. We tested whether Nivolumab treatment had an impact on the expansion of inducible Treg populations in our in vitro system. We found that, on average, Nivolumab treatment significantly increased the percentage of CD4^+^CD25^+^CD127^-/low^FoxP3^+^ Treg cells. However, the effects of Nivolumab treatment were relatively modest, with an average fold change of only 1.22 compared to the control (Figure 6). 

## 3. Discussion

Identification of predictive biomarkers for anti-PD-1 therapy is a major challenge in this type of immunotherapy and, in general, for any immune modulated anti-cancer therapies. Most studies involving the identification of biomarkers for anti-PD-1 therapy were conducted retrospectively, in which T cell functions of patients, such as cytokine productions in serum, were assessed by comparing these functions before or after receiving anti-PD-1 therapy [18,22,24]. In our study, we propose a robust and reliable non-invasive assay using easily accessible tissues from patients by using PBMCs as a source of test materials for prospectively identifying new biomarkers. We used an anti-CD3 mAb to provide TCR signaling for baseline T cell activation, which can also induce PD-1 expression on T cells. This PD-1 expression is a requirement for the effects of Nivolumab (Supplementary Figure S1), since the main mechanism of action of anti-PD-1 therapy is blocking the interaction between PD-1 on T cells and PD-L1 on tumor cells or stromal cells [26]. Furthermore, in our PBMC testing system, the anti-CD3 mAb also significantly increases expression of PD-L1 in non-T cells (Supplementary Figure S2A), which may play a similar role as PD-L1 expressed on tumor cells or stromal cells within the tumor microenvironment. In addition, unlike testing T cell responses to anti-PD-1 therapy using SEB activated T cells, which only test T cell responses in TCR V chain-positive T cells, an anti-CD3 mAb can provide baseline T cell activation for all T cells.

Our results suggest that data from a PBMC based assay can reflect and recapitulate many of the changes that occur in patients treated with anti-PD-1 therapy. These findings include: 1) the cytokine responses to Nivolumab are highly heterogeneous among donors. No two donors show an identical cytokine response to Nivolumab treatment among all tested donor PBMCs. Some donors demonstrated a tendency to respond better in terms of specific cytokines induced by Nivolumab: the maximum number of cytokines produced by Nivolumab treatment is eight cytokines in donor 7. In contrast, three donors’ PBMCs did not respond at all in terms of induction of cytokine expression induced by Nivolumab (donors 2, 5, and 21) (Supplementary Table S2). These heterogeneous responses are consistent with the heterogeneous responses seen in patients undergoing treatment with anti-PD-1/PD-L1 therapy. We also found that the individual cytokine response to Nivolumab is highly heterogeneous. Nivolumab increases IL-9 production by twofold in eight donors, but fails to increase IL-6, IL-1α, and IL-1RA to a similar extent in any of the tested donors. This diversity in cytokine responses provides additional challenges for the use of a single cytokine as a predictive biomarker. Development of an algorithm that can capture the complexity of the cytokine response to anti-PD-1/PD-L1 therapy is needed if using cytokine production as the biomarker. 2) Nivolumab can also increase the expansion of inducible regulatory T cells, which is consistent with previous work demonstrating that Pembrolizumab treatment increases the expansion of these regulatory T cells [13], and that the increased expansion of Tregs is associated with a poor prognosis for Nivolumab treatment in cancer patients. Of note, flow cytometric analyses indicate that Nivolumab does not have an impact on expression levels of T cell activation markers, or the functional marker Granzyme B, as was found in a dendritic cell and T cell co-culture MLR system [27]. Therefore, our data indicate that the results gained from flow cytometry assays might not be suitable for use as biomarkers using our PBMC model. These differences may be due to the level of PD-L1 expression on dendritic cells being much higher than on PBMCs [34] (Supplementary Figure S2). Supporting this, the magnitude of elevated cytokine production is also less profound than that observed in the dendritic cell and T cell co-culture system [27].

Our data demonstrate that anti-PD-1 treatment can have potent and profound effects on many T cell functionalities that were previously unappreciated. For example, we found that Nivolumab increases production of the Th9 associated cytokine IL-9 by more than two-fold in more than half of the donors (Figure 2). Accumulating evidence indicates that Th9 cells are important components for anti-tumor immunity responses [35]. A recent study demonstrated that Nivolumab treatment increases the Th9 cell frequency in metastatic melanoma patients who respond to treatment, suggesting that Th9 cell responses could potentially be used as a predictive biomarker of Nivolumab treatment efficacy [36]. Our study further elucidates the potential role of Th9 cells in anti-PD-1 mediated anti-cancer immunity, suggesting that further studies investigating the biological effects of Th9 in Nivolumab mediated anti-cancer immune responses could potentially lead to a new combination therapy that targets Th9 cells. In addition, we found that Nivolumab significantly increases the production of the Th17 associated cytokines IL-17A, IL-21, and IL-22, but not IL-23. Indeed, unpublished data indicate that Th17-associated cytokines can modulate Nivolumab-induced T cell responses. In addition, the elevation of IL-17 production has been shown to occur in patients undergoing treatment with Nivolumab [37], further suggesting that our model system may reflect the underlying mechanisms of action of anti-PD-1 therapy.

Our study provides rational and supportive evidence for using a PBMC-based assay that may lead to the identification of novel predictive biomarkers for anti-PD-1 therapy. However, many challenges remain. First, although healthy PBMCs have been used to characterize the functionality of anti-PD-1 therapies such as Nivolumab and Pembrolizumab, it will be important to determine whether the heterogeneous cytokine responses we have observed occur in PBMCs derived from cancer patients undergoing treatment with anti-PD-1 therapy. It would also be critical to determine whether the elevated cytokine production we have observed positively correlates with cancer types that respond well to anti-PD-1 therapy, such as lung cancer and melanoma, but does not correlate with cancer types that do not respond to anti-PD-1 therapy, such as prostate and pancreatic cancers. 

Second, can such an assay be useful in reflecting the complexity of the tumor microenvironment? It is well established that the tumor microenvironment can shape anti-tumor T cell responses. It will be interesting to see how our PBMC system compares with a model using tumor cells or tumor stromal cells such as Tregs and macrophages. Such a system might overcome the difficulty in acquiring clinical samples at the initial stages of study. 

Third, recent studies indicate that the mechanism of anti-PD-1 therapy occurs not only through modulating T cell responses, but also potentially through modulating NK cell and macrophage functions [38,39]. It will be informative to see whether our model system can also recapitulate these changes. It is unclear if an algorithm can be developed that would be able to capture the complexity of the cytokine response to anti-PD-1 therapy. It will be important to determine whether combining our current observations regarding elevated cytokine production with currently existing biomarker(s) such as PD-L1 or neoantigen expression can synergize to improve the accuracy of treatment and disease outcomes.

In summary, although questions need to be addressed before considering the clinical validation of this work, our findings demonstrate that a simple PBMC based approach provides the potential for identifying novel biomarkers, as well as facilitates our understanding of the mechanisms of anti-PD-1 therapy, which could be used to develop novel combination therapies with anti-PD-1 therapy [40].

## 4. Materials and Methods

### 4.1. Reagents 

Nivolumab (Bristol-Myers Squibb, NJ), an IgG4 monoclonal antibody (mAb) against PD-1, was purchased through the NIH Pharmacy (Bethesda, MD). Fluorescence-conjugated anti-human PD-1 (clone EH12.1), CD14 (MϕΠ9), CD3 (OKT3), CD4 (RPA-T4), CD8 (RPA-T4), CD25 (2A3), CD69 (FN50), CD127 (hIL-7R-M21), Granzyme B (GB11), Ki67 (B56), and IFN-γ (4S-B3) were all purchased from BD Biosciences (San Jose, CA, USA). Fluorescence-conjugated anti-human PD-L1 (clone 130021) was from R&D Systems (Minneapolis, MN, USA). GolgiPlug protein transport inhibitor (containing Brefeldin A) and cytokine fixation/permeabilization solution kit were also from BD Biosciences. Anti-human CD3 monoclonal antibody (clone OKT3) was from BioLegend (San Diego, CA, USA). FoxP3 staining kit and permeabilization buffer, and multiplex bead-based cytokine kits were purchased from Thermo Fisher Scientific (Waltham, MA, USA). Fetal bovine serum and human AB serum were purchased from Valley Biomedical (Winchester, VA, USA).

### 4.2. Cell Culture

Human peripheral blood mononuclear cells (PBMCs) were obtained from healthy human donors. All donors provided written informed consent, and all methods were carried out in accordance with established and relevant guidelines and regulations. The protocol for obtaining and using these tissues was reviewed and approved by both the National Institutes of Health (the Human Research Protection Program, or HRPP) and the Food and Drug Administration (the Research Involving Human Subjects Committee, or RIHSC) internal review boards.

Human PBMCs were obtained from healthy human volunteers by leukapheresis and purified from whole blood by ficoll-hypaque sedimentation. Cells were frozen at a concentration of 50 × 10^6^ in 1 mL freezing medium containing 90% fetal bovine serum (Valley Biomedical, VA) and 10% DMSO (Sigma-Aldrich, St. Louis, MO, USA) until needed. A total of 30 donors’ PBMCs were used in this study. The demographic information about each donor is provided in Supplementary Materials Table S3.

For cell culture, 5 × 10^5^ PBMCs were incubated in 200 μL complete RPMI1640 medium containing 5% human AB serum, 1% Penicillin/Streptomycin, 1% Hepes, and 0.1 mM 2-mercaptoethanol in the presence of a sub-optimal concentration of anti-human CD3 mAb (0.1 μg/mL) with Nivolumab (10 μg/mL) or without. After three days in culture, the media were harvested for multiplex cytokine analysis, and the cells were harvested for flow cytometry analyses.

### 4.3. Cytokine Luminex Assay

Cytokine assays were performed in duplicate using multiplex bead-based kits for the indicated human cytokines per the manufacturers’ instructions (ThermoFisher Scientific, CA). A total of 23 cytokines were routinely assessed in this study: IL-1α, IL-1β, IL-1RA, IL-2, IL-4, IL5, IL-6, IL-7, IL-9, IL-12, IL-13, IL-15, IL-17, IL-18, IL-21, IL-22, IL-23, IL-27, IFN-α, IFN-γ, TNF-α, TNF-β, and GM-CSF. Fluorescence of beads was measured using a Luminex Bioplex 200 analyzer (Bio-Rad Laboratories, Hercules, CA, USA), and data analysis was performed using the BioPlex Manager software (BioHercules, CA. USA) based on a five-parametric logistic nonlinear regression curve-fitting algorithm. All data were reviewed and validated by two independent investigators.

### 4.4. Flow Cytometry

#### 4.4.1. Intracellular Staining for Ki67, Granzyme B, and FoxP3

For multicolor staining, cells were harvested and washed with DPBS buffer. Cells were first stained with Live/Dead Aqua dye (Invitrogen, Carlsbad, CA, USA) according to the manufacturer’s instructions, followed by two washes with DPBS containing 5% fetal bovine serum and 0.1% sodium azide. Cells were then fixed and permeabilized using FoxP3 Fixation/Permeabilization solution (ThermoFisher Scientific) at 4 °C for one hour and washed twice with 1× permeabilization buffer (ThermoFisher Scientific). Cells were then stained with a mixed antibody cocktail containing mAbs to CD14, CD3, CD25, CD69, CD4, CD8, CD127, Granzyme B, and Ki67, as well as FoxP3 for 30 min. at 4 °C. Cells were then washed twice with 1× permeabilization buffer and resuspended in DPBS containing 5% fetal bovine serum and 0.1% sodium azide for flow cytometry analyses.

#### 4.4.2. Intracellular Staining for Cytokines

For intracellular cytokine staining, cells were cultured as described above in the presence of GolgiPlug for 4 h. Cells were then harvested for staining for the surface markers as well as for intracellular IFN-γ and TNF-α using cell fixation/permeabilization solutions (BD Biosciences) per the manufacturer’s instructions. All samples were assessed, and the data were acquired using a five-laser BDLSR FortessaTM flow cytometry system (BD Biosciences). Data were analyzed with FlowJo software (Version 10.7.1, BD Biosciences). 

### 4.5. Statistical Analysis

Statistical analysis was performed using GraphPad Prism software (version 7, GraphPad Software, San Diego, CA, USA). A two-tailed Student’s *t*-test was used to analyze differences in cytokine production and expression of T cell activation markers among the experimental groups. A *p*-value of < 0.05 was considered statistically significant. 

## Supplementary Materials

Supplementary materials can be found at https://www.mdpi.com/1422-0067/21/23/9023/s1.
