# Supplementary material for: Exploring the Potential Use of a PBMC-Based Functional Assay to Identify Predictive Biomarkers for Anti-PD-1 Immunotherapy
Source: Int J Mol Sci. 2020 Nov 27;21(23):9023. doi: 10.3390/ijms21239023 (PMC7730837; doi:10.3390/ijms21239023)
Supplement: Supplementary file 1 [file ijms-21-09023-s001.pdf]

Supplementary Materials

Exploring the Potential Use of a PBMC-Based Functional Assay to Identify Predictive Biomarkers for Anti-PD-1 Immunotherapy

Silvia M. Bacot, Taylor A. Harper, Rebecca L. Matthews, Christie Jane Fennell, Adovi Akue, Mark A. KuKuruga, Shiowjen Lee, Tao Wang and Gerald M. Feldman

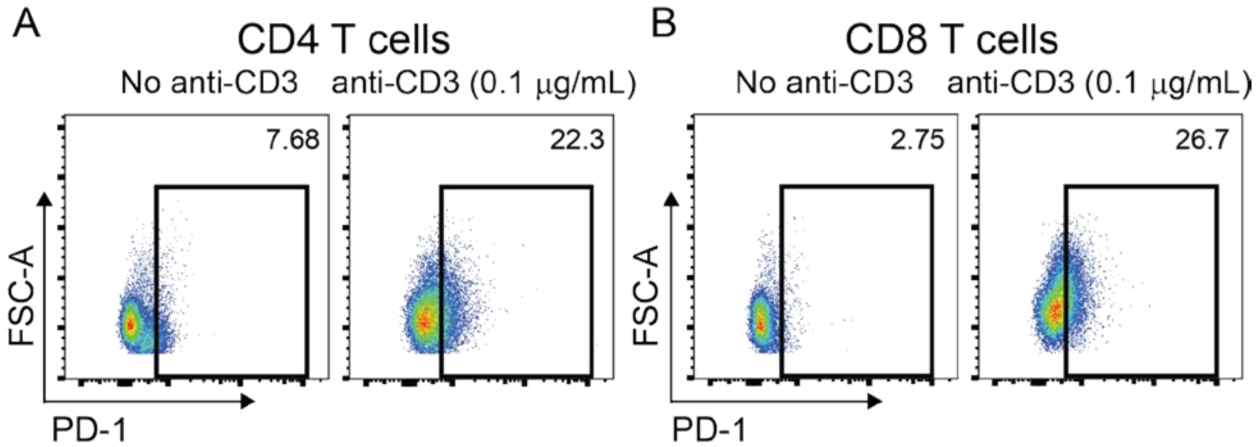

Supplementary Figure S1. Anti-CD3 mAb upregulates the expression of PD-1 in CD4 and CD8 T cells. (**A,B**) Frozen PBMCs were thawed and cultured in RPMI1640 medium containing 5% AB human serum in the presence or absence of anti-CD3 mAb (0.1 μg/mL) for three days. Cells were harvested for flow cytometry analysis of expression of PD-1 on T cells and PD-L1 on non-T cells. Shown is the expression of PD-1 on CD4+ T cells (**A**) and CD8+ T cells (**B**) of one representative donor among 16 healthy donors.

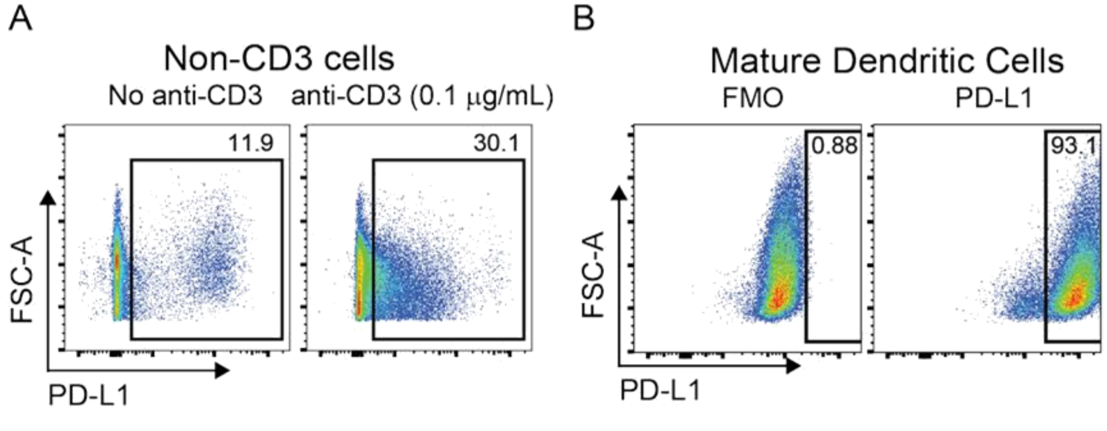

Supplementary Figure S2. Expression of PD-L1 on non-CD3 cells and mature dendritic cells. (A) Frozen PBMCs were thawed and cultured in RPMI1640 medium containing 5% AB human serum in the presence or absence of anti-CD3 mAb (0.1 μg/mL) for three days. Cells were harvested for flow cytometric analysis of expression of PD-L1 on CD3 negative cells. Shown is the expression of PD-1 on CD3 negative cells of one representative donor among eight healthy donors. **(B)** The method of generation of mature dendritic cells

was described in our previous publication.1 Briefly, monocytes were incubated for six days in complete RPMI 1640 medium containing 50 ng/mL GM-CSF and 20 ng/mL IL-4, and then were stimulated with 100 ng/mL LPS for 24 h for maturation. Mature dendritic cells were then harvested for flow cytometry analysis of expression of PD-L1. Fluorescence Minus One (FMO) Controls were used to determine the positive/negative interface (left panel). Shown is the expression of PD-L1 of one representative donor among four healthy donors (right panel).

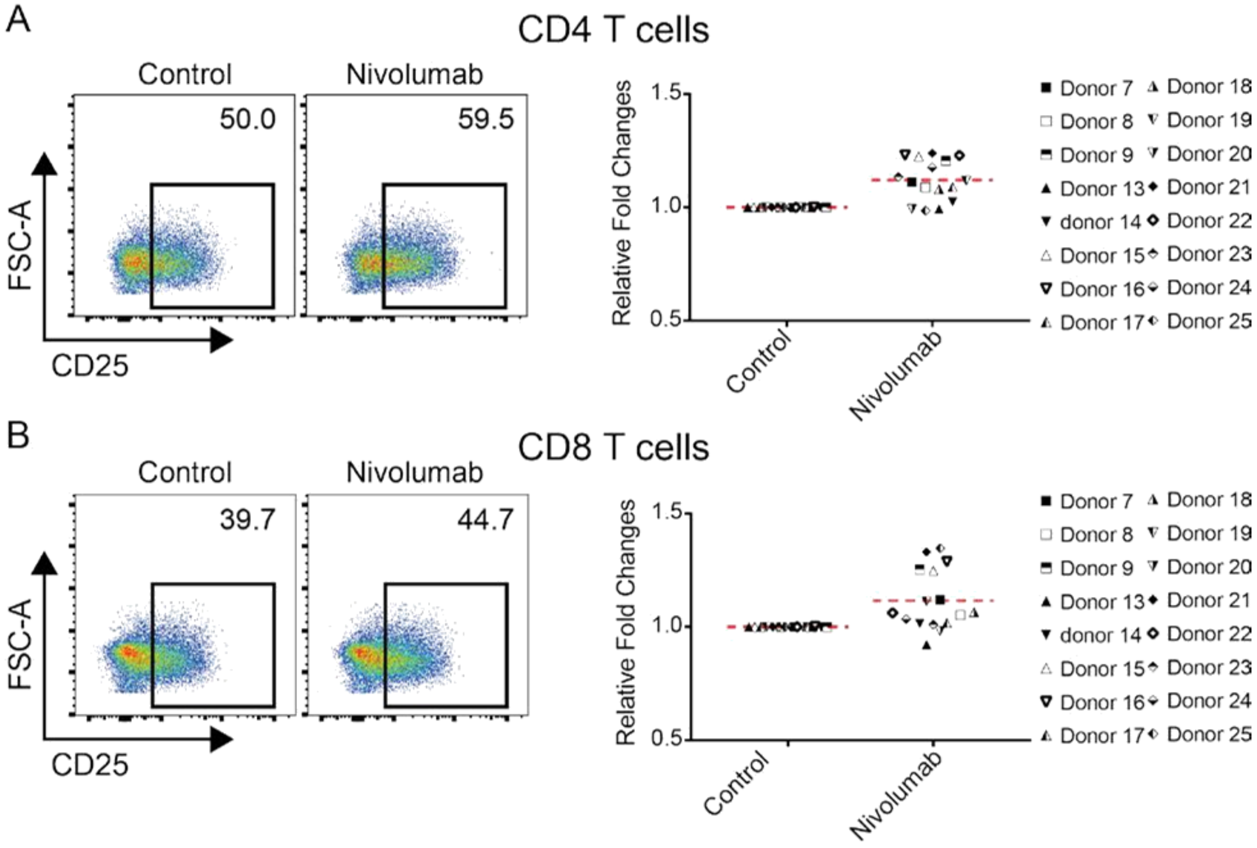

Supplementary Figure S3. Nivolumab treatment does not impact T cell activation. PBMCs were treated as described in **Figure 1** and were then harvested for flow cytometric analysis of the expression of T cell activation marker CD25 in CD4+ T cells **(A)** and CD8+ T cells **(B)**. Left panels show the data from one representative donor, whereas the right panel provides a summary of data from different sixteen individual donors.

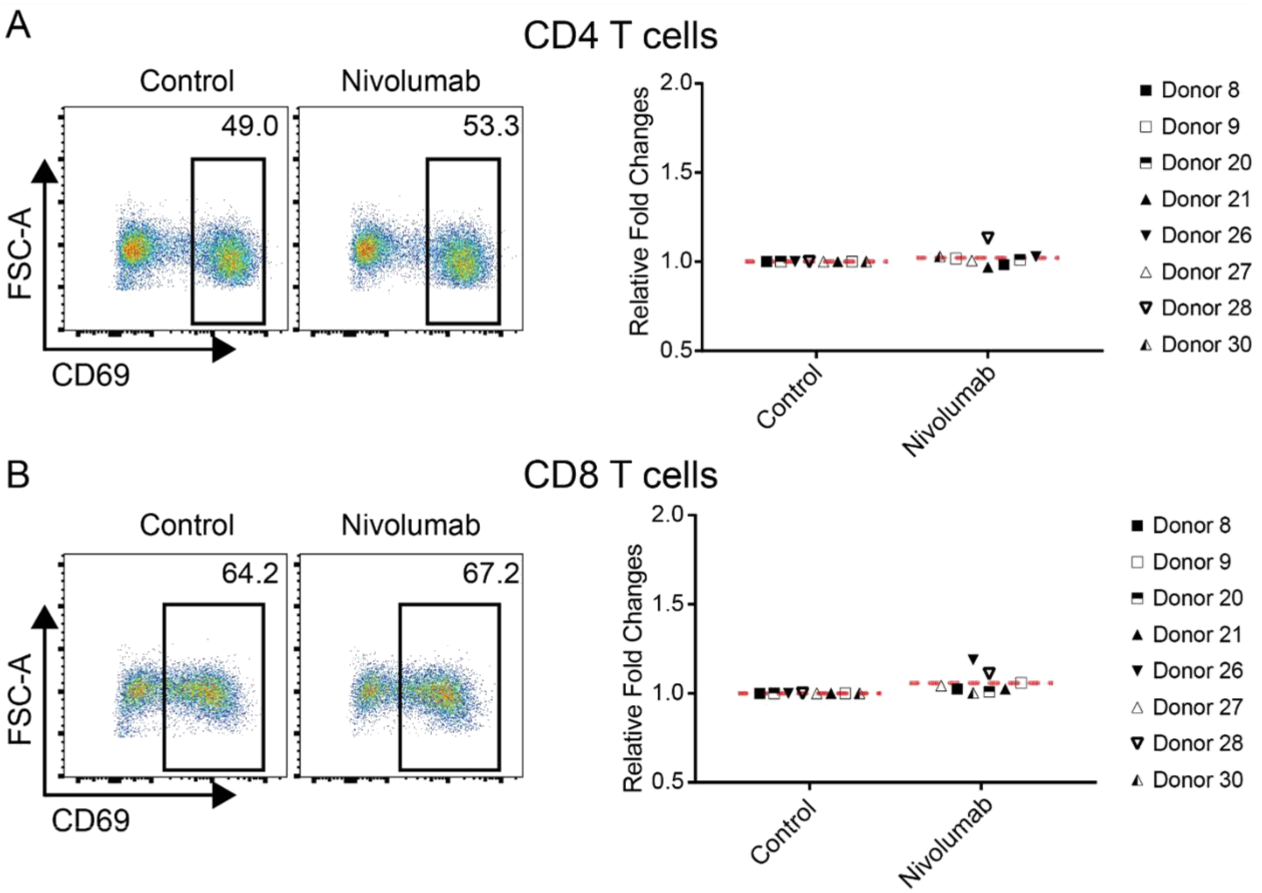

Supplementary Figure S4. Nivolumab treatment does not impact expression of T cell activation marker CD69. (**A**,**B**) Frozen PBMCs were thawed and cultured in RPMI1640 medium containing 5% AB human serum. Cells were then treated with Nivolumab (20 μg/mL) in the presence of anti-CD3 mAb (0.1 μg/mL) for eighteen hours, and were then harvested for flow cytometric analysis of the expression of T cell activation marker CD69 in CD4+ T cells **(A)** and CD8+ T cells **(B)**. Left panels show the data from one representative donor, whereas the right panel provides a summary of data from different eight individual donors.

Supplementary Table S1. Nivolumab does not increase Th1-associated cytokine productions in the absence of anti-CD3 mAb (0.1 μg/mL).

**Study ID**	**IFN-γ (pg/mL)**		**IL-2 (pg/mL)**		**TNF-α (pg/mL)**		**TNF-β (pg/mL)**		**IL18 (pg/mL)**		**GM-CSF (pg/mL)**	
	Pre-Rx	Post- Rx	Pre-Rx	Post-Rx	Pre-Rx	Post-Rx	Pre-Rx	Post- Rx	Pre-Rx	Post-Rx	Pre-Rx	Post- Rx
Donor 1	OOR <	OOR <	9.7	8.3	4.76	5.62	OOR <	OOR <	OOR <	OOR <	OOR <	OOR <
Donor 6	21.28	21.48	49.07	58.15	235	186	8.75	12.9	45.36	46.71	59.51	86.4
Donor 13	OOR <	OOR <	OOR <	OOR <	OOR <	OOR <	35.42	38.3	OOR <	OOR <	OOR <	OOR <
Donor 14	OOR <	OOR <	OOR <	OOR <	*1.73	OOR <	54.78	61.09	OOR <	OOR <	OOR <	OOR <
Donor 21	OOR <	OOR <	OOR <	OOR <	*7.2	*2.59	85.53	87.56	OOR <	OOR <	*0.98	*0.98

*Value = Value extrapolated beyond standard range. OOR< = Out of Range Below. Rx = treatment

Supplementary Table S2.

Donor Number	Treatment	IFN-r	IL-2	TNFa	IL-10	GM-CSF	IL-1b	IL-12	IL-6	IL-4	IL5	IL-17A	IL-21	IL-22	IL-23	IL-27	IL-9	IFNa	IL-1A	IL-1RA	IL-13	IL-18	TNFb
Donor 1	Control	5083.18	59.95	434.24	151.05	387.91	421.54	OOR <	12490.1	368.17	109.53	91.28	20.03	OOR <	OOR <	OOR <	88.96	4.79	40.49	117352.4	715.12	106.29	290.92
	Nivolumab	10443.68	63.24	556.94	351.61	532.68	740.29	7.12	16778.29	358.04	97.52	89.05	64.31	OOR <	OOR <	OOR <	132.2	3.83	51.45	81127.82	518.29	137.51	352.77
Donor 2	Control	10758.79	61.82	640.61	298.22	228.1	5475.1	27.11	26731.48	176.25	56.63	42.54	52.14	OOR <	OOR <	OOR <	25.48	2.7	108.76	41323.74	51.87	161.89	95.8
	Nivolumab	10188.2	72.23	694.8	288.32	306.87	4328.81	28.99	27071.93	187.8	56.63	59.94	59.64	OOR <	OOR <	OOR <	22.62	OOR <	93.05	37920.83	51.87	162.72	106.15
Donor 3	Control	10950.59	46.82	468.76	362.19	703.51	51.27	11.77	6192.55	162.48	87.39	60.13	116.58	53.87	*0.25	OOR <	222.68	OOR <	7.76	47321.52	410.39	122.86	346.81
	Nivolumab	10305.9	79.69	648	464.52	1129.24	116.35	21.01	9341.07	224.36	95.66	78.34	157.82	42.8	OOR <	OOR <	284.64	OOR <	11.71	48189.43	389.86	125.84	419.48
Donor 4	Control	930.87	53.84	677.6	177.59	888.6	92.55	OOR <	5851.63	217.16	409.78	206.91	36.65	OOR <	OOR <	OOR <	403.3	OOR <	13.98	91102.75	5405.46	46.72	289.24
	Nivolumab	4391.28	57.46	626.28	411.65	1155.61	132.05	OOR <	8175.6	281.7	320.97	213.89	50.84	OOR <	OOR<	OOR <	347.76	OOR <	14.86	32250.87	4266.3	97.59	299.17
Donor 5	Control	1527.86	370.74	2934.35	4160.46	3517.67	874.82	12.8	8994.48	327	106.27	1892.76	241.99	1609.5	27.18	65.32	385.44	2.94	220.2	25427.81	1639.72	564.15	1094.29
	Nivolumab	1423.35	376.84	3826.81	5162.5	5704.11	1071.58	8.61	6788.46	490.16	77.63	1417.27	406.71	1672.42	19.76	111.27	697.7	0.48	183.32	31719.94	1315.61	544.48	1400.27
Donor 6	Control	1038.37	89.07	7810.3	2444.9	5048.84	2198.29	*2.95	10274.94	199.54	311.25	3027.77	317.69	1452.18	*15.17	119.54	1082.05	1.1	271.97	51072.87	2900.48	893.9	1402.49
	Nivolumab	965.41	138.93	11297.08	4143.51	8727.73	4035.2	*3.08	9687.84	369.02	242.49	3443.64	569.26	2185.31	23.1	152.46	2323.53	4.47	332.73	67454.64	2779.93	723.94	1787.05
Donor 7	Control	1178.98	298.57	1557.35	367.55	299.16	930.36	1.91	2282.95	86.36	24.01	67.1	9.06	70.43	*7.08	*4.59	24.91	3.89	173.45	32352.5	332.6	240.69	185.54
	Nivolumab	1441.21	60.3	2730.93	1084.07	1791.69	1729.41	*1.13	2330.9	176.29	40.58	308.55	44.65	188.91	*7.68	18.08	125.81	1.39	128.53	39282.13	1066.51	439.64	579.58
Donor 8	Control	15902.65	29.89	1219.4	763.24	247.05	1516.48	OOR <	62077.3	68.95	128.58	77.43	47.46	13.58	OOR <	OOR <	110.22	18.4	62.98	62075.93	843.71	363.02	226.79
	Nivolumab	16670.43	43.56	1482.16	1189.59	530.34	2867.46	OOR <	31589.37	128.06	129.3	62.58	67.74	27.52	OOR <	OOR <	168.52	7.28	88.63	59123.03	818.17	381.15	238.15
Donor 9	Control	3389.54	44.02	732.65	256.76	1291.95	171.33	OOR <	3860.47	245.48	281.41	32.29	163.13	OOR <	OOR <	OOR <	286.05	OOR <	22.05	13039.77	945.55	271.68	220
	Nivolumab	2911.38	139.2	1302.14	618.01	2216.19	277.5	OOR <	4045.69	594.95	729.33	60.86	226.97	OOR <	OOR <	OOR <	617.47	OOR <	29.13	12645.13	1525.52	298.29	316.79
Donor 10	Control	23614.44	31.91	1773.75	697.93	1492.43	657.28	OOR <	144182.15	97.44	146.48	740.21	261.73	478.96	OOR <	OOR <	685.9	OOR <	43.86	61637.45	829.48	408.71	360.53
	Nivolumab	24247.68	58.12	3706.19	2145.01	2864.38	1106.01	OOR <	133649.36	203.57	324.76	1729.79	390.27	344.72	OOR <	OOR <	1018.81	0.82	62.9	58038.61	1498.13	438.06	500.69
Donor 11	Control	28672.79	30.4	1992.14	1005.13	922.15	2081.82	OOR <	OOR >	81.63	251.82	486.17	332.81	70.13	OOR <	*6.05	527.21	8.98	44.11	62362.09	1782.21	484.91	484.11
	Nivolumab	22523.81	38.94	3902	1470.05	2013.98	6039.63	8.6	OOR >	158.98	269.5	299.44	501.56	98.88	OOR <	*6.05	603.89	4.84	62.51	69891.18	2196.9	598.89	569.34
Donor 12	Control	1266.06	9.54	58.56	300.32	24.25	598.73	OOR <	OOR >	*5.26	8.31	486.8	8.34	52.84	*2.50	OOR <	12.51	N/A	N/A	N/A	57.58	63.14	N/A
	Nivolumab	2085.88	10.03	108.95	1003.42	37.86	911.72	OOR <	OOR >	*7.34	11.77	698.82	15.8	87.32	*2.70	OOR <	28.71	N/A	N/A	N/A	119.32	80.53	N/A
Donor 13	Control	5907.08	59.13	1758.5	105.98	377.59	57.47	44.27	1480.09	25.71	43.29	77.53	107.57	338.54	41.74	OOR <	25.97	OOR <	3.45	14420	177.13	91.31	1305.78
	Nivolumab	5379.01	136.84	2359.81	245.94	525.5	78.36	125.1	1950.93	45.11	35.83	143.31	247.22	365.33	61.73	OOR <	37.29	OOR <	4.84	12534.65	183.79	88.64	1515.62
Donor 14	Control	7515.94	35.07	3693.47	450.41	775.06	215.64	31.36	8093.21	76.33	91.48	170.94	194.41	199.81	32	OOR <	571.44	OOR <	26.72	24709.38	646.47	100.76	2030.08
	Nivolumab	6808.36	59.78	4836.44	985.74	1145.88	280.37	65.54	8627.26	103.38	103.33	354.39	398.96	299.52	39.78	*5.89	777.59	OOR <	40.22	19993.65	595.09	98.1	2364.37
Donor 15	Control	5482.45	17.68	1397.13	118.28	724.91	254.47	*2.97	2999.34	58.66	127.92	88.95	37	112.22	30.72	OOR <	46.97	OOR <	24.17	18543.27	390.34	85	671.62
	Nivolumab	6482.75	19.42	1637.06	249.16	956.65	286.65	*4.04	2751.62	80.33	109.17	107.61	63.5	135.39	43.05	OOR <	108.6	OOR <	23.73	16453.9	407.59	94.71	839.4
Donor 16	Control	3238.03	12.49	799.65	53.96	453.32	135.38	*1.53	3950.53	20.01	40.99	22.99	12.82	113.59	25.63	OOR <	13.71	OOR <	6.86	15484.8	228.96	58.92	388.88
	Nivolumab	5009.72	12.49	1039.64	140.64	625.51	166.73	*4.06	3493.61	28.53	54.91	34.25	19.6	142.17	36.52	OOR <	33.2	OOR <	8.65	13719.14	236.93	73.75	528.79
Donor 17	Control	5991.44	57.47	2457.46	144.61	663.06	124.69	60.5	2719.52	26.56	59.26	102.63	127.28	393.83	63.76	OOR <	33.2	OOR <	4.47	15748.61	208.99	86.46	1593.12
	Nivolumab	6059.48	162.17	3662.17	350.57	838.1	150.74	211.6	3525.51	43.75	49.61	231.83	431.13	538.29	108.96	OOR <	51.88	OOR <	7.93	12396.75	253.65	92.29	2074.94
Donor 18	Control	5404.38	22.66	3931.52	349.27	1946.99	1246.58	*0.66	10744.24	29.09	494.84	280.78	75.91	240.5	*15.69	*1.42	1701.43	OOR <	136.71	66551.93	3038.47	109.69	1717.75
	Nivolumab	6500.7	25.19	4823.5	968.48	2323.96	2383.6	*1.18	15440.27	38.53	436.67	397.4	112.63	326.41	19.38	*5.89	2567.28	OOR <	171.8	72409.99	2644.17	122.45	1964.17

Donor 19	Control	4707.83	9.38	1687.05	30.02	1255.42	433.26	*1.35	4495.98	106.02	115.73	54.95	15.25	42.53	*10.28	OOR <	37.97	*0.34	38.11	76682.95	1811.46	96.4	730.9
	Nivolumab	5695.31	21.07	3634.92	87.84	2250.4	723.92	*2.46	6220.84	454.75	227.49	119.3	33.02	68.37	*10.87	OOR <	97.76	*0.28	71.73	74248.74	3779.6	119.81	1370.73
Donor 20	Control	4252.38	12.49	1994.99	210.78	824.68	678.91	*1.07	*218302.53	72.07	91.09	210.63	33.71	91.96	*14.47	OOR <	63.63	OOR <	46.97	58930.03	427.99	80.12	769.55
	Nivolumab	3876.44	19.98	3182.92	720.71	1655.94	929.16	*0.43	*53249.81	163.27	114.57	206.01	52.76	129.51	*9.69	OOR <	138.43	OOR <	75.6	55836.36	887.1	81.1	972.16
Donor 21	Control	7158	65.58	3603.5	205.66	778.01	428.18	*4.82	4946.68	88.29	141.07	449.12	114.44	188.1	33.29	OOR <	799.79	OOR <	38.97	29072.26	601.92	104.38	1734.81
	Nivolumab	6666.56	79.87	3375.89	599.09	753.88	591.31	*3.14	6405.75	104.44	98.23	689.16	196.91	221.02	26.9	OOR <	1115.88	OOR <	60.19	28979.17	502.01	88.16	1539.79

OOR: Out of range. OOR >: Out of range above. OOR <: Out of range below. *Value: Value extrapolated beyond standard range.

Supplementary Table S3. Demographic information of all donors for the study.

Donor ID	Age	Sex	Race	Study ID
W092115050185	42	Female	Caucasian	Donor 1
W092115050764	66	Male	Caucasian	Donor 2
W092115050924	41	Male	African American	Donor 3
W092115050965	58	Male	Caucasian	Donor 4
N/A	47	Male	African American	Donor 5
W092115051182	31	Female	African American	Donor 6
W092116050816	21	Male	Caucasian	Donor 7
W092116050851	68	Male	African American	Donor 8
W092115051016	43	Male	Hispanic	Donor 9
W092115051435	32	male	Caucasian	Donor 10
W092115051506	23	Female	Caucasian	Donor 11
W092116050884	24	Female	Caucasian	Donor 12
W092116051083	58	Female	African American	Donor 13
W092116051093	69	Male	African American	Donor 14
W092116 05070	49	Male	Caucasian	Donor 15
W092118050945	29	Female	Caucasian	Donor 16
W092116 850803	47	Female	Caucasian	Donor 17
W092115051265	64	Male	Caucasian	Donor 18
W092115051355	40	Male	Caucasian	Donor 19
W092115051596	47	Male	African American	Donor 20
W092116 050975	55	Male	African American	Donor 21
W092118050797	32	Female	African American	Donor 22
W092118051047	23	Male	Caucasian	Donor 23
W092116050803	47	Female	Caucasian	Donor 24
W092116050750	29	Male	African American	Donor 25
W092119014150	36	Female	Caucasian	Donor 26
W092119014451	59	Male	African American	Donor 27
W092119014716	25	Female	African American	Donor 28
W092116050702	49	Male	Caucasian	Donor 29
W092119014966	N/A	N/A	N/A	Donor 30

N/A: not available.

References

1. 1. Yue, P.; Harper, T.; Bacot, S.M.; Chowdhury, M.; Lee, S.; Akue, A.; Kukuruga, M.; Wang, T.; Feldman, G.M. BRAF and MEK inhibitors differentially affect nivolumab-induced T cell activation by modulating the TCR and AKT signaling pathways. *OncoImmunology* **2018**, *8*, e1512456, doi:10.1080/2162402x.2018.1512456.
